# Estimating Biomass and Carbon Sequestration Capacity of *Phragmites australis* Using Remote Sensing and Growth Dynamics Modeling: A Case Study in Beijing Hanshiqiao Wetland Nature Reserve, China

**DOI:** 10.3390/s22093141

**Published:** 2022-04-20

**Authors:** Siyuan Wang, Sida Li, Shaoyan Zheng, Weilun Gao, Yong Zhang, Bo Cao, Baoshan Cui, Dongdong Shao

**Affiliations:** 1 State Key Laboratory of Water Environmental Simulation, School of Environment, Beijing Normal University, Beijing 100875, China; 201911180138@mail.bnu.edu.cn (S.W.); sidali_lsd@foxmail.com (S.L.); shaoyanzheng@mail.bnu.edu.cn (S.Z.); wl.gao@mail.bnu.edu.cn (W.G.); cb@bnu.edu.cn (B.C.); cuibs@bnu.edu.cn (B.C.); 2 Research and Development Center for Watershed Environmental Eco-Engineering, Beijing Normal University, Zhuhai 519087, China; 3 Hanshiqiao Wetland Nature Reserve Management Office, Beijing 101399, China; hdong@path.org

**Keywords:** *Phragmites australis*, urban wetlands, growth dynamics model, remote sensing, biomass, carbon sequestration

## Abstract

Estimating the biomass of *Phragmites australis* (Cav.) Trin. ex Steud., i.e., a common wetland macrophyte, and the associated carbon sequestration capacity has attracted increasing attention. Hanshiqiao Wetland Nature Reserve (HWNR) is a large *P. australis* wetland in Beijing, China, and provides an ideal case study site for such purpose in an urban setting. In this study, an existing *P. australis* growth dynamics model was adapted to estimate the plant biomass, which was in turn converted to the associated carbon sequestration capacity in the HWNR throughout a typical year. To account for local differences, the modeling parameters were calibrated against the above-ground biomass (AGB) of *P. australis* retrieved from hyperspectral images of the study site. We also analyzed the sensitivity of the modeling parameters and the influence of environmental factors, particularly the nutrient availability, on the growth dynamics and carbon sequestration capacity of *P. australis*. Our results show that the maximum AGB and below-ground biomass (BGB) of *P. australis* in the HWNR are 2.93 × 10^3^ and 2.49 × 10^3^ g m^−2^, respectively, which are higher than the reported level from nearby sites with similar latitudes, presumably due to the relatively high nutrient availability and more suitable inundation conditions in the HWNR. The annual carbon sequestration capacity of *P. australis* in the HWNR was estimated to be 2040.73 gC m^−2^ yr^−1^, which was also found to be highly dependent on nutrient availability, with a 50% increase (decrease) in the constant of the nutrient availability *K_NP_*, resulting in a 12% increase (23% decrease) in the annual carbon sequestration capacity. This implies that a comprehensive management of urban wetlands that often encounter eutrophication problems to synergize the effects of nutrient control and carbon sequestration is worth considering in future practices.

## 1. Introduction

*Phragmites australis* is a typical emergent wetland plant and a globally distributed species [1]. *P. australis* plays a crucial role in maintaining the ecological function of wetlands and providing a variety of ecosystem services, such as water purification, habitat provision, biodiversity conservation and carbon sequestration [2,3,4,5,6]. Among these critical functions, *P. australis* can assimilate considerable carbon through photosynthesis during its growth cycle, which can effectively mitigate carbon emission and support carbon neutrality [7]. Biomass, i.e., the dry weight of the living organic component, of *P. australis* is a key predictor of its carbon sequestration capacity [8]. Under growing concern of climate change due to excessive carbon emission, estimation of the biomass and carbon sequestration capacity of wetland plants, including *P. australis*, has received increasing attention to support carbon management and achieve the goal of carbon neutrality [9,10,11].

Plant biomass is traditionally measured through field sampling on plot scale, which is both time-consuming and labor-intensive, and is often prohibited due to site accessibility issues [12]. In addition to field sampling, a vegetation growth dynamics model has also been developed to estimate the biomass. Growth dynamics models are established based on the relevant eco-physiological processes of the modelled plants, such as photosynthesis, respiration, and mortality, which allow measuring the plant biomass with higher temporal resolution (e.g., daily) and thus understanding the variation trend of biomass and carbon sequestration capacity in greater detail [13,14,15]. Specifically, Asaeda and Karunaratne [14] were among the pioneers who first established a growth dynamics model of *P. australis* by selecting five state variables (biomass of shoots, inflorescence, roots, old rhizomes, and new rhizomes) to simulate the plant growth cycle. Plant growth was quantified using mathematical relationships based on the integral effects of photosynthesis, respiration, mortality, and translocation of materials between different organs (e.g., above- and below-ground parts) to calculate the biomass change, which can well reflect the growth dynamics of *P. australis* [7,14]. By specific calibration and validation, the model has been applied to explore the growth dynamics of *P. australis* in different case studies [7,16]. For instance, Soetaert et al. [7] adopted the model to examine the meteorological effects on the growth trend of *P. australis* in the Scheldt estuary, and Eid et al. [16] applied the model to simulate the biomass and carbon sink of *P. australis* in a Mediterranean eutrophic lake. In addition, as the impact of environmental factors (e.g., latitude and nutrient availability) on the plant growth traits, including phenological points and biomass, was parameterized in the growth dynamics models [13,17,18], the response of plant biomass and carbon sequestration capacity to the variation in environmental factors can be further simulated, which is beneficial for carbon management under environmental changes [17]. It is worth noting that, despite the versatility and flexibility of the plant growth dynamics models, their development and application still rely on the availability of field biomass data for the necessary model calibration and validation [19].

Remote sensing data, such as optical images, light detection and ranging (LiDAR) point cloud data, and synthetic aperture radar (SAR) data, have been widely used to quantify plant above-ground biomass (AGB) [20,21,22,23]. Based on the relationship between remote sensing data (e.g., reflectance, vegetation indices) and the field-collected actual biomass, remote sensing technology can help estimate AGB rapidly and accurately [23,24]. It is particularly helpful in the context of wetlands due to their typically remote and inaccessible locations and expansive area [25]. For example, Luo et al. [26] proved the ability of hyperspectral compact airborne spectrographic imager (CASI) data for retrieving the AGB of *P. australis* in the Zhangye National Wetland Park, China, and Du et al. [27] established the empirical model for retrieving the AGB of *P. australis* in Dafeng, China. Therefore, plant biomass retrieved by remote sensing can be used as a potential data source for calibrating and validating plant growth dynamics models.

Located in the northeast of Beijing Municipality, the Hanshiqiao Wetland Nature Reserve (HWNR) is a typical marsh and the largest *P. australis* wetland in the city. With a core area mainly occupied by *P. australis* [28], the HWNR provides important ecological services for the city, such as biodiversity conservation [29], eutrophication regulation [30], and air purification [31]. As the capital and a major metropolitan city in China, Beijing is facing growing pressure for mitigating carbon emission, and how to achieve its goal of carbon neutrality has become a critical issue [32]. Therefore, an understanding of the growth trend and carbon sequestration capacity of *P. australis* in the HWNR to help assess its potential use as a major carbon sink for the city can effectively support the carbon management of the city. To this end, we adapted the growth dynamics model of *P. australis* originally developed by Asaeda and Karunaratne [14] to estimate the biomass (including the AGB and below-ground biomass (BGB)) and carbon sequestration capacity of *P. australis* in the core area of the HWNR in this study. Hyperspectral remote sensing images, together with an empirical retrieval equation proposed by Li et al. [33] for the HWNR, were used to obtain the plant biomass data for model calibration. The temporal variation in biomass and carbon sequestration capacity of *P. australis* throughout its growth cycle in the HWNR were simulated and analyzed, and the effects of the key environmental factors on the phenological points and maximum AGB were further discussed. The specific objectives of this study were as follows: (1) to establish a growth dynamics model that is calibrated against the hyperspectral image data to estimate the AGB and BGB of *P. australis* in the HWNR; (2) to illustrate the variation trend throughout the growth cycle of *P. australis* in the HWNR; and (3) to analyze the influence of environmental factors on the biomass and carbon sequestration capacity of *P. australis* in the HWNR. To accomplish these objectives, data and methods used in the present study, including the introduction of the study area are documented in Section 2. The detailed modelling results are presented in Section 3, and then Section 4 discusses the effects of the key environmental factors on plant growth and carbon sequestration capacity as well as the implications for wetland management. Finally, the main conclusions are drawn in Section 5.

## 2. Methods

### 2.1. Study Site

**Figure 1:**
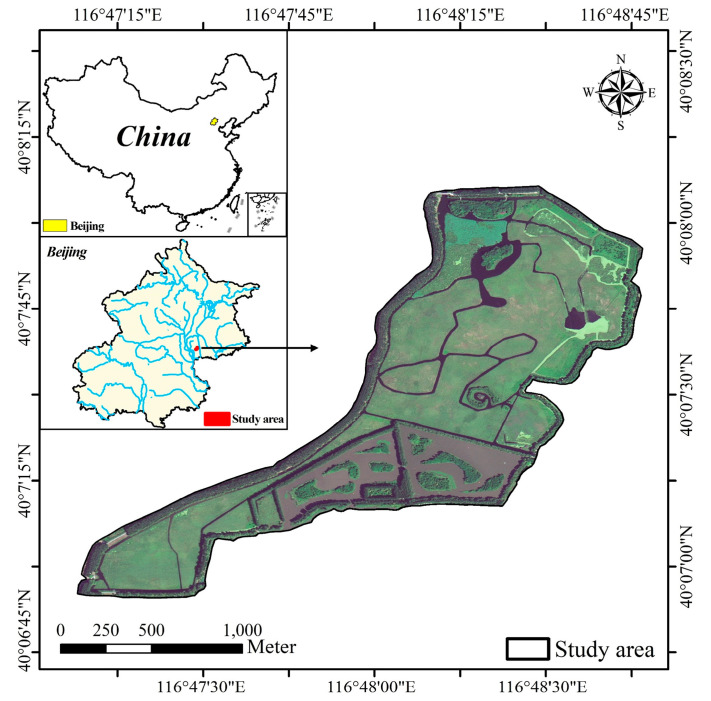
Location of the core area of the Hanshiqiao Wetland Nature Reserve (HWNR).

The Hanshiqiao Wetland Nature Reserve (HWNR) (40°06′ N, 116°48′ E) is located in Shunyi District, Beijing, China. The Reserve, which was established by the Beijing Municipal Government in April 2005, is a typical urban wetland in the North China Plain. The HWNR has a total area of 1900 ha and comprises a core area of 163.5 ha, which was chosen as our study area (Figure 1).

The Reserve is located in the Chaobai River catchment, and the Caijia River flows through it and becomes its main natural water source. This region has a semi-humid continental monsoon climate, with an annual average temperature of 11.8 °C and annual precipitation of 644 mm, mainly in July and August [28]. There are 182 species of birds and 222 species of plants belonging to 144 genera and 55 families recorded in the Reserve at present. Macrophytes such as *P. australis* and *Typha orientalis* are widely distributed, and *P. australis* is the dominant species [28].

### 2.2. Imagery Dataset

Hyperspectral images were acquired for *P. australis* interpretation in the study area, retrieving its AGB. *P. australis* usually starts growing in March and reaches its maximum biomass in July [34] and undergoes a controlled harvest by the administration agency of the HWNR in late November. Thus, images of 1 November 2020, 17 June 2021, and 31 July 2021 were obtained from the Orbita hyperspectral satellite constellation (OHS) (https://www.obtdata.com/, accessed on 12 October 2021) to reflect the conditions of *P. australis* at the peak and the end of the growth cycle.

Developed by Zhuhai Orbita Aerospace Science & Technology Co. Ltd. (Zhuhai, China), the OHS is the first hyperspectral satellite constellation in the world with surface coating technology for sensors [35]. The constellation consists of eight hyperspectral satellites, and the first batch of four satellites was successfully launched on 26 April 2018, followed by the second batch of four satellites launched on 19 September 2019. Satellites of OHS have an on-orbit life span of more than 5 years, and the revisit cycle is 5 days. A dual-frequency global navigation satellite system (GNSS) receiver (GPS and Beidou) is used to measure the position of OHS in its orbit. The sensor altitude of OHS is around 500 km above the ground, resulting in images of a spatial resolution of 10 m. Each satellite of the OHS has 256 bands with a wavelength ranging from 400 nm to 1000 nm at approximately 2.5 nm intervals. However, only 32 bands can be selected due to limitations in data transmission and storage. The main parameters of OHS are listed in Table 1 [36].

High-resolution images (pixel size < 10 m) such as SPOT images with a pixel size of 2.5 m and QuickBird images with a pixel size of 2.4 m have been widely used to form a dataset of training samples for *P. australis* interpretation, as they contain abundant spatial information [37,38]. In this study, a high-resolution BJ-2 image of June 2021 was used for selecting training samples for interpretation. The BJ-2 images are provided by the BJ-2 satellite constellation, which was developed by Surrey Satellite Technology Ltd. (https://www.sstl.co.uk/, accessed on 2 August 2021) and launched in July 2015. The constellation has a revisit cycle of one day and provides sub-meter resolution images of red, green, and blue bands at a pixel size of 0.8 m and infrared images at a pixel size of 3.2 m. With a relatively high spatial resolution, BJ-2 images have been utilized for various applications such as monitoring landslides, extracting hydraulic engineering constructions, classifying land-cover types, etc. [39,40,41]. In this study, the 0.8 m resolution BJ-2 image that contained red, green, and blue bands was acquired, geo-rectified to the WGS 84 datum (50N), and further used as a reference image for the registration of the OHS images.

### 2.3. P. australis Growth Dynamics Model

#### 2.3.1. Model Formulation

The present model was adapted from the *P. australis* growth model originally developed by Asaeda and Karunaratne [14]. Similarly, the biomass of *P. australis* (g DM m^−2^) was divided into above-ground and below-ground organs. The above-ground organs were further subdivided into flowering shoots including fertile leaves and inflorescences, as well as non-flowering shoots including (sterile) leaves and non-flowering secondary shoots. The below-ground organs were subdivided into roots and new and old rhizomes. The changes in biomass of each organ throughout the growth cycle were estimated by incorporating the net growth in each organ as a function of photosynthesis, respiration, mortality, and inter-organ translocation. Briefly, the biomass of each organ was calculated as follows:

(1) $$B=P-R-M+T_{r},$$

where *B*, *P*, *R*, *M,* and *T_r_* denote biomass, gross photosynthesis production, respiration loss, mortality biomass, and inter-organ translocation, respectively.

**Figure 2:**
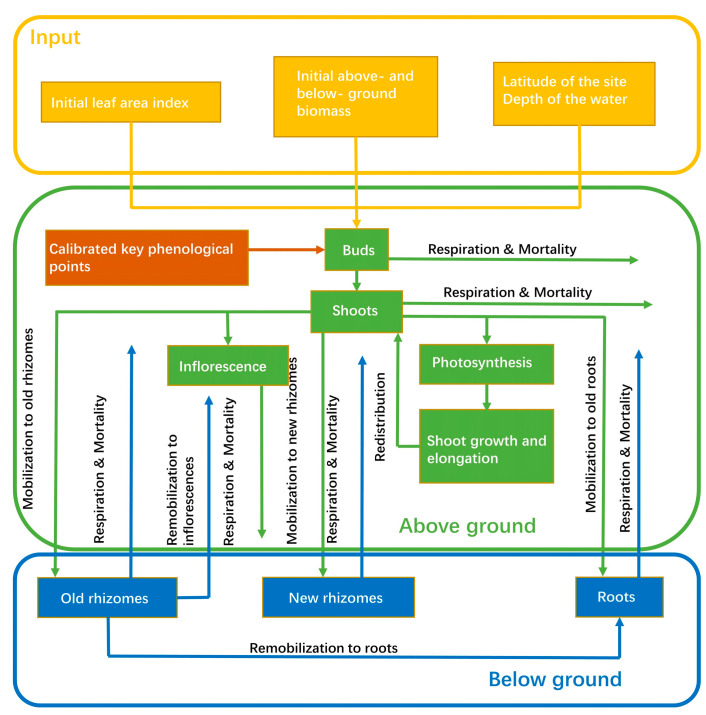
The structure of the *Phragmites australis* growth model (adapted from Zheng et al. [17]).

The specific framework of the model is shown in Figure 2. The model stratified the above-ground part into 1 cm thick horizontal layers, where the net growth budget and shoot elongation were calculated separately. Based on the above organ division, we selected five state variables—i.e., biomass of shoots, inflorescences, roots, old rhizomes and new rhizomes—to characterize plant growth. The details of the growth equations for each state variable are documented in the Supplementary Materials. The growth equations for each layer and organ were solved simultaneously using the fourth-order Runge–Kutta method, and the time step of the iteration was set to one day.

The growth dynamics model reflects the biomass change during a typical growth cycle of *P. australis* and neglects interannual variation. The major phenological points and the corresponding phenological events in the present model are summarized in Table 2. As the phenological points are largely correlated with latitude and further modulated by local conditions such as meteorology and terrain, the empirical regression equations that describe the relationship among the phenological points of *P. australis* derived by Asaeda and Karunaratne [14] through reported case studies from multiple countries (see Supplementary Materials) were used as the basis for further calibration, as shown in the following. All dates used to describe phenological points were Julian days.

#### 2.3.2. Model Calibration

Following Asaeda and Karunaratne [14], the key parameters (see Supplementary Materials) in the *P. australis* growth dynamics model include parameters that are related to the generic ecophysiology of *P. australis* (e.g., half saturation constant of age for shoot photosynthesis) and parameters that are more dependent on local conditions and are thus highly site-specific (e.g., the phenological points). Generic parameters adopted values from the original model and needed no calibration. Site-specific parameters needed to be further calibrated using field data.

In general, observation of the occurrence of most phenological events is difficult or sometimes impossible. Among those phenological events, the start of plant growth is relatively easier to detect [42]. Once the date when growth began as denoted by *t_b_* is specified, the date when panicles start forming (*t_f_*), the date when the upward translocation from old rhizomes terminates (*t_e_*) and the date that marks the onset of senescence (*t_s_*) can be preliminarily derived using the respective empirical relationships of the various phenological points as functions of *t_b_*. Further calibration and the resulting phenological points of *P. australis* in the HWNR are shown in Section 3.2.

To determine the order of calibration of the remaining site-specific parameters, their influence on the model predictions of the AGB and BGB were assessed by carrying out a sensitivity analysis, in which a shifting range of ±50% for each parameter was implemented one at a time. The most sensitive modeling parameters thus identified were then calibrated one at a time through comparison against remote sensing retrieved biomass data by trial and error. The values of the parameters associated with the least sums of squared residuals between model predictions and retrieved data were adopted as the calibrated values, as presented in Section 3.

#### 2.3.3. Model Execution

To carry out the simulation, input data such as the latitude were specified. Regarding the initial values, we assumed the initial leaf area index (LAI) to be zero as a normal practice in previous studies [17,33,43,44]. Since *P. australis* in the study area are harvested at the end of each year, the initial AGB was assumed to be zero as well. A series of simulations assuming different initial BGB values were conducted, and the value associated with the least mean square error between the predicted AGB in a year and the retrieved AGB was adopted. The model was implemented in MATLAB R2020b (MathWorks, Natick, MA, USA).

### 2.4. P. australis Interpretation and AGB Retrieval

#### 2.4.1. Preprocessing of Hyperspectral Images

Radiometric calibration and atmospheric correction were carried out on the OHS images to obtain surface reflectance required by subsequent analyses. Radiometric calibration was performed to convert the original digital number (DN) values into apparent radiance using the following formula [45]:

(2) $$Rad=gain\times DN\;/\;\left(TDI\;Stage\right)+offset,$$

where *Rad* is the apparent radiance, *gain* refers to the gain coefficient of the absolute radiometric calibration, *TDI Stage* is a parameter that describes how active pixels are placed in a time-delay-integration (TDI) image that is provided by the instrument supplier, and *offset* is the absolute radiometric calibration offset coefficient. Atmospheric correction was performed using the fast line-of-sight atmospheric analysis of spectral hypercubes (FLAASH) module [46] to obtain surface reflectance with Rad. Images with surface reflectance were registered to the corresponding BJ-2 image with the registration root mean square error (RMSE) less than 0.5 pixel. The registered OHS images were resampled to their original pixel size (10 m) by the nearest-neighbor resampling technique [47]. Radiometric calibration, atmospheric correction, and registration were performed using ENVI 5.3 (L3Harris Technologies, Broomfield, CO, USA), and resampling of the registered OHS images was carried out using ArcGIS 10.3 (ESRI, Redlands, CA, USA).

#### 2.4.2. Interpretation of *P. australis*

In this study, as the plant growth model is a generic zero-dimensional plot-scale model, the potential inter-annual variation in the spatial distribution of *P. australis* in the study area was neglected and *P. australis* was interpretated using the OHS hyperspectral image of June 2021, as the plant was matured at that time. A total of 344 polygon samples of water body and vegetation were randomly selected from the high-resolution BJ-2 image. As *Nymphaea tetragona* is the dominant floating plant and *T. orientalis* is the most common coexistent species of *P. australis* in the HWNR [48,49], vegetation samples were further categorized into *P. australis*, *N. tetragona*, *T. orientalis,* and other vegetation (Figure S1), resulting in five classes in total. The polygon samples were divided into two groups. One group consisting of 237 training samples was used for interpretation, and the other group consisting of 107 validation samples was used for evaluating the accuracy of interpretation (Table 3).

Separability metrics including Jeffries–Matusita distance (JM) and the transformed divergence (TD) were adopted to evaluate the efficiency of the selected training samples [50]. The values of these two metrics generally range from 0 to 2, and the corresponding separability is considered to be good if the metric value is greater than 1.9.

Support vector machine (SVM) was used to extract *P. australis* in the study area. SVM is an advanced non-parametric machine learning classifier that has been proved to be robust and reliable for *P. australis* interpretation using hyperspectral images [51]. This method follows the idea of inputting samples into a high-dimensional space and constructing a hyperplane to separate samples into different classes, and a separation margin defined by the hyperplane indicates the difference between two classes of samples, with the optimal hyperplane that can maximize the margin selected to generate the ultimate separation result [52]. With the advantage that fewer training samples are generally required, SVM also demonstrates better adaptability and faster training speed than other machine learning methods [53].

In the present study, the radial basis function (RBF) kernel was selected for SVM, as it has been proven to be more accurate than other kernels [54]. Following relevant previous studies [55], the two key parameters of SVM—i.e., the value of gamma in RBF kernel and penalty parameter—were set as 0.01 and 100, respectively. Overall accuracy (OA) and Kappa coefficients were calculated to assess the accuracy of interpretation results. Producer’s accuracy (PA) and user’s accuracy (UA) were also calculated to estimate the degree of misclassification for each class, as PA and UA represent the percentage of correct interpretation results in the validation samples and the training samples pixel by pixel [56]. Based on the feature information revealed by the high-resolution BJ-2 image, artificial visual interpretation was also performed on the results of SVM to correct the misclassified pixels and obtain the final interpretation result. All the steps described in this section were carried out in ENVI 5.3 (L3Harris Technologies, Broomfield, CO, USA).

#### 2.4.3. Retrieval of *P. australis* AGB

The AGB of *P. australis* was retrieved using an empirical model constructed by Li et al. [33]. The model was developed based on the observed AGB data and a canopy spectrum of *P. australis* in the HWNR. The coefficient of determination (R^2^) of the model was 0.72, which indicates that the retrieval model was reasonably reliable. The specific equation is as follows:

(3) AGB =−15.859x+4.8574,

where *x* represents the maximum surface reflectance in the wavelength range of 510–560 nm. Selection of the maximum surface reflectance and the calculation were performed in ArcGIS 10.3 (ESRI, Redlands, CA, USA).

### 2.5. Evaluation of Carbon Sequestration Capacity

The carbon sequestration capacity of *P. australis* in the study area was calculated based on the photosynthesis equation [57]:

(4) $$6{\mathrm{CO}}_{2}+6H_{2}O\;\to \;6O_{2}+C_{6}{\;H}_{12}O_{6}$$

The ratio between the molecular weight of the organic material (starch) and CO_2_ sequestered by photosynthesis was 1:1.62, which means per 1 g of organic material fixated approximately 1.62 g CO_2_ (0.44 g C). The net primary production (NPP) was calculated to represent the net accumulation of organic material generated via photosynthesis. NPP is defined as the amount of total organic material minus the loss of material caused by plant respiration [58]. As shown in Section 2.3.1, the net growth (i.e., living biomass) in the model was defined as the production of photosynthesis minus the loss due to respiration and mortality. Hence, in the present study, daily NPP (g m^−2^) was estimated as follows:

(5) $$\mathrm{NPP}=\Delta B_{t}+M_{t},$$

where Δ*B_t_* represents the variation in simulated living biomass between two adjacent days (i.e., *B_t_* and *B_t−1_*), and *M_t_* refers to the simulated mortality biomass of day *t*. Thereafter, daily carbon sequestration capacity (gC m^−2^) was estimated using the calculated NPP and the above ratio. Monthly and annual carbon sequestration capacities were further obtained by adding up daily carbon sequestration capacities throughout each month and the entire year, respectively. The total annual carbon sequestration capacity of *P. australis* in the HWNR was calculated by multiplying the annual carbon sequestration capacity per square meter with the distribution area of *P. australis* in the HWNR.

## 3. Results

### 3.1. Distribution and Retrieved AGB of P. australis

**Figure 3:**
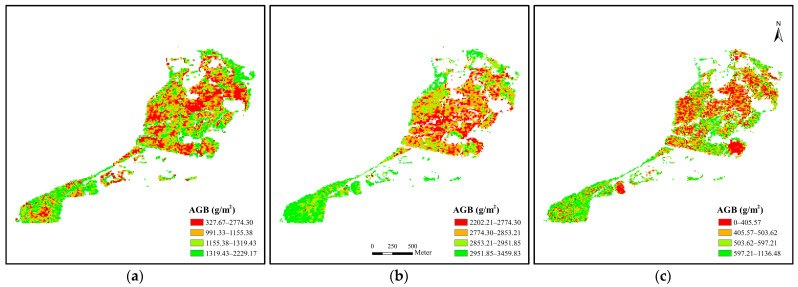
Map of retrieved above-ground biomass (AGB) of *P. australis* in three representative months: (**a**) June, (**b**) July, and (**c**) November.

As shown in Table 4, the training samples used in the present study were reliable, as the JM and TD values between *P. australis* and other classes were consistently higher than 1.9. Table 5 shows the accuracy assessment results of the interpretation. With an overall accuracy of 92.21% and a Kappa coefficient of 0.88, the accuracy of the interpretation result was satisfactory. According to the producer’s accuracy and user’s accuracy, the error of interpretation mainly arose from the difficulty in identifying *N. tetragona* and *T. orientalis*. Producer’s accuracy showed that pixels of *N. tetragona* and *T. orientalis* were mainly misclassified as water body (165/895) and *P. australis* (194/694), respectively. Fairly good results had been achieved for *P. australis* interpretation, with the producer’s accuracy and user’s accuracy attaining 94.71% and 96.95%, respectively. The misclassified pixels were manually corrected, resulting in the distribution of *P. australis* shown in Figure 3, with a total distribution area of 86.58 ha.

Figure 3 shows the retrieved results and the distribution pattern of the AGB of *P. australis* in the study area. Among the three representative months of June, July, and November, *P. australis* in July had the highest averaged AGB (2863.59 ± 131.86 g m^−2^), followed by June (1156.72 ± 247.09 g m^−2^) and November (498.38 ± 145.04 g m^−2^). A statistical analysis on the pixel numbers was further performed, and we defined the pixels ranged between lower and upper quartiles as the concentrated range of the AGB. Results show that AGB for June, July, and November mainly concentrated between 991.33 and 1319.43 g m^−2^, 2774.30 and 2951.85 g m^−2^, and 405.57 and 597.21 g m^−2^, respectively. The above results show a consistent trend that *P. australis* kept growing until it experienced mortality in November. Areas with high AGB values were mainly concentrated in the southern part of the study area throughout the three months. On the contrary, low AGB values were prevalent in the northern part.

### 3.2. Calibration and Sensitivity Analysis of Growth Dynamics Model

**Figure 4:**
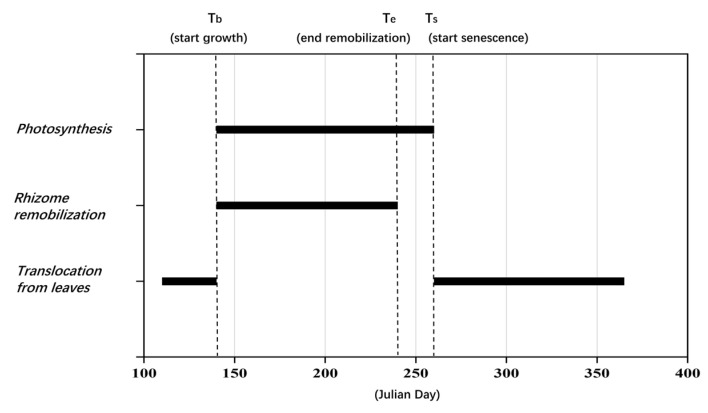
Phenological events as implemented in the *P. australis* model.

According to the retrieved AGB, upward translocation of biomass to the above-ground organs was observed at the end of April, then the AGB reached the peak value around August and finally declined to the minimum around November. To reflect the typical growth pattern and make the model more suitable for simulating the growth cycle of *P. australis* in the HWNR, the following calibration of the key parameters was performed:

(1) The dates of the phenological points were calibrated based on the preliminary value obtained by the empirical relationships in Section 2.3.2. Most phenological points were moved backward to match the actual growth cycle of *P. australis* in the HWNR. The specific time of some representative phenological events during the growth cycle of *P. australis* is shown in Figure 4.

(2) The constant of the nutrient availability and the biomass transfer rate from fertile leaf, sterile leaf, and peduncle to new rhizomes were slightly increased, and the respiration rate of above-ground organs was reduced in our model, to achieve higher peak biomass, as shown by the retrieved results.
(3) The mortality rates of the above-ground organs at the onset of senescence, especially those of shoots and flowers, were increased after *t_s_* to match the occurrence of abrupt biomass loss at that stage.

**Figure 5:**
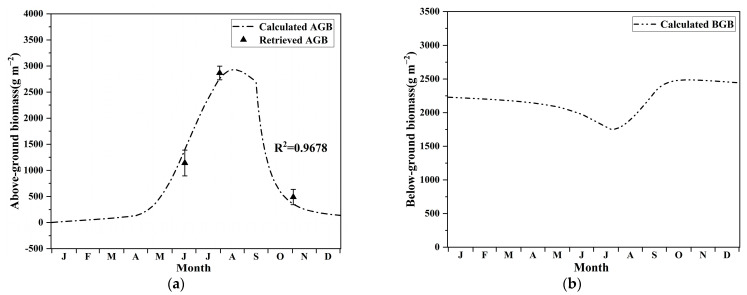
Seasonal biomass variation in *P. australis*: (**a**) in the above-ground organs; (**b**) in the below-ground organs.

The seasonal variation in the predicted AGB of *P. australis* was compared with the average value of the retrieved data across the study area (Figure 5a). For all retrieved data points, the model predictions agreed satisfactorily (R^2^ = 0.9678) and captured the general growth pattern.

For instance, a mildly increasing biomass growth rate in the early-growing season, followed by a drastically increasing biomass growth rate during the mid-growing season, the decline in biomass due to senescence at the end of the growing season, the time of peak biomass, etc., were all successfully reproduced by our model prediction, which was consistent with the growth pattern of *P. australis* reported in the literature [14]. Figure 5b shows the seasonal variation in the BGB of *P. australis*, which was in line with the typical growth pattern of the below-ground organs—e.g., the reduction in the BGB during the early growing season due to the supply of stored material to shoot growth—and the increase in the BGB after *t_p_* (new rhizomes start forming) because of the downward translocation of material from photosynthesis and shoot dry matter, etc.[14]. Additionally, the BGB at the end of the growth season was slightly higher than that at the beginning, which can be attributed to the need of nutrient storage and preparation for growth in the following year [17].

Through sensitivity analysis of the key modeling parameters, the growth dynamics model was found to be most sensitive to maximum specific net daily photosynthesis rate, specific mortality rate of shoots, fraction of shoot transfer to rhizome, constant of the nutrient availability, and fraction of shoot biomass for elongation. Table 6 shows the sensitivity of the model output on key modelling parameters for the study site. For instance, a 50% increase in the maximum specific net daily photosynthesis rate resulted in a 56.35% increase in the AGB at *t_s_* (the date when shoots start senescence), and a 50% decrease resulted in a 58.01% decrease in the AGB at *t_s_*. By contrast, a 50% increase or decrease in the other four parameters led to much lesser effects on the AGB at *t_s_*. In addition, the outcome of the model was also affected marginally by some other parameters such as respiration rates, mortality rates, and fractions of transfer, etc.

### 3.3. Estimation of Carbon Sequestration Capacity

As shown in Figure 5, living AGB and BGB of *P. australis* ranged from 1.87 to 2.93 × 10^3^ g m^−2^ and 1.75 × 10^3^ to 2.49 × 10^3^ g m^−2^, respectively. In addition, mortality biomass ranged from 0 to 203.95 g m^−2^ and 0.07 to 0.62 g m^−2^ for the above- and below-ground part, respectively. Therefore, daily carbon sequestration capacity for the above- and below-ground part varied in the range of −0.05 to 19.71 gC m^−2^ and −2.58 to 6.67 gC m^−2^, respectively. Maximum daily carbon sequestration capacity was attained on the 207th day. Annual carbon sequestration capacity was 2040.73 gC m^−2^ yr^−1^, which translated to a total amount of 1766.86 tC being sequestrated annually by *P. australis* in the HWNR.

**Figure 6:**
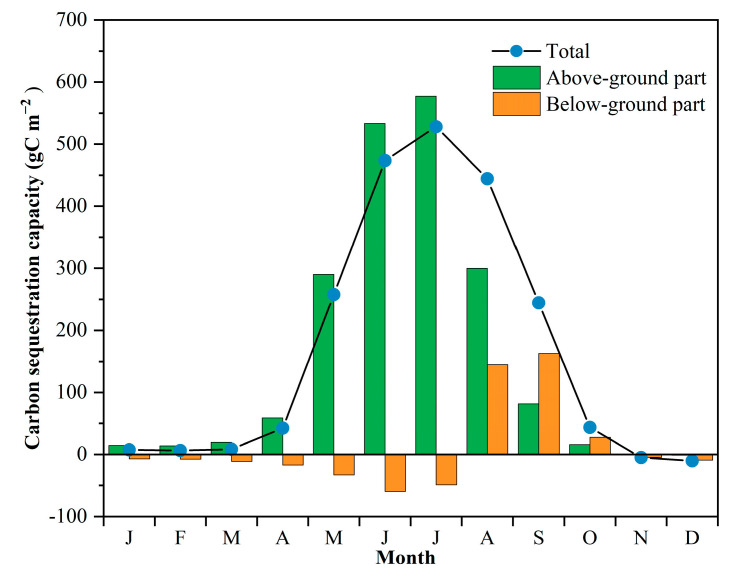
The variation in monthly carbon sequestration capacity of *P. australis* in the Hanshiqiao Wetland Nature Reserve.

As shown in Figure 6, monthly whole-plant carbon sequestration capacity was found to slowly increase from January to April, which was consistent with the phenological pattern that the above-ground part of *P. australis* started growing at the end of April (120th day). Growth of sterile shoot in May led to a rapid increase in the AGB and monthly whole-plant carbon sequestration capacity, i.e., 509% higher than that in April. The monthly whole-plant carbon sequestration capacity reached its peak in July with the value of 528 gC m^−2^, which was mainly contributed by the largest monthly carbon sequestration capacity of the above-ground part in July of 577.19 gC m^−2^.

As new peduncles and roots started to form from the end of July (205th day), below-ground carbon sequestration capacity increased afterwards. Daily below-ground carbon sequestration capacity became positive from the 207th day, and monthly carbon sequestration capacity of the below-ground part became higher than that of the above-ground part in September. As both the above- and below-ground part continued senesce from the middle of September (260th day), monthly whole-plant carbon sequestration capacity kept decreasing and became negative in November and December.

## 4. Discussion

### 4.1. Comparison in Phenological Points and Biomass of P. australis from Other Sites

**Figure 7:**
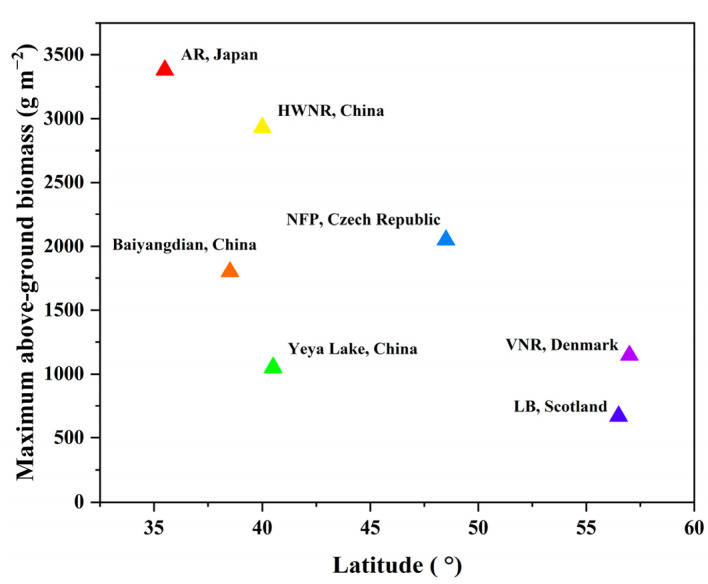
The maximum AGB of *P. australis* in various sites from different latitudes.

The biomass and phenological points of *P. australis* from four additional sites were collected from the literature to compare with the results in the HWNR (Table 7). Compared with *P. australis* from higher-latitude area (e.g., Nesyt fish pond, Czech Republic, and Loch of Balgavies, Scotland), the maximum AGB of *P. australis* in the HWNR was larger, and its start dates of shoot growth and panicle formation were relatively later, which was consistent with the general pattern of latitudinal variation reported by Karunaratne et al. [59] and Clevering et al. [60] (Figure 7). However, significant variability can also be found between biomass of *P. australis* in the HWNR and those in similar latitudes, such as Baiyangdian Lake (Hebei, China) [61] and Yeya Lake wetland (Beijing, China) [62], which indicated that biomass is also affected by other confounding local factors, such as nutrient level [63,64], inundation depth, and hydroperiod (i.e., inundation frequency and duration) [65,66]. This variability can be found in other latitudes as well, e.g., *P. australis* in Loch of Balgavies, Scotland, and Vejlerne Nature Reserve, Denmark—two sites with similar latitudes that also have significant differences in biomass [67,68].

Figure 7 shows that the maximum AGB of *P. australis* in the HWNR was considerably higher than that in Baiyangdian Lake (Hebei, China) [61,65] and Yeya Lake wetland (Beijing, China) [62], which can be attributed to the influence of nutrients and inundation depth. Wang et al. [70] studied the optimum inundation depth for *P. australis* in different growing stages and found that a relatively low inundation depth (≤0.10 m) can facilitate the germination and establishment of *P. australis* at the early stage (from the end of April to May) and that higher inundation depth is more suitable at the rapid growth stage (from the end of June to the end of August). Due to the annual precipitation and evaporation pattern [28], a prominent seasonal variation trend with lower inundation depth in spring and higher inundation depth in summer was exhibited in the HWNR, which was consistent with the general requirement of suitable inundation depth for *P. australis* [70] and contributed to the relatively high biomass of *P. australis* in the HWNR.

In addition, previous studies have shown that higher nutrient concentration facilitates the growth of *P. australis* [71,72]. Comparing the nutrient concentration between the HWNR and Yeya Lake wetland, the annual average values of total nitrogen and total phosphorus in the former (1.93 mg/L and 0.33 mg/L) were significantly higher than those in the latter (1.59 mg/L and 0.067 mg/L) [73], which can also lead to the higher maximum ABG of *P. australis* in the HWNR.

### 4.2. Influence of Nutrient Availability on Growth and Carbon Sequestration of P. australis

**Figure 8:**
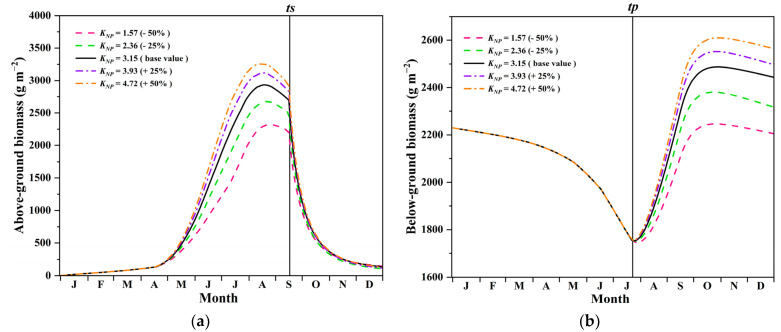
Biomass variation in *P. australis* subject to scenarios with varying nutrient availability: (**a**) in the above-ground organs; (**b**) in the below-ground organs. *t_s_*: the date when shoots start senescence; *t_p_*: the date when peduncles and new rhizomes start forming.

Our results show that the constant of the nutrient availability (*K_NP_*) was one of the key environmental factors of the growth dynamics model (Table 6), which can further influence the carbon sequestration capacity. As eutrophication poses potential threat in the HWNR [74], we further explored how it influences the growth and carbon sequestration capacity of *P. australis* by setting five scenarios with different constant of the nutrient availability. Assuming the calibrated *K_NP_* in Section 3.2 as the base value, Scenario 1 was set to the base scenario, while Scenarios 2–5 were set by varying *K_NP_* at intervals (−50%, −25%, +25%, +50%). Figure 8a,b shows simulated results of the AGB and BGB in all five scenarios.

With higher values of *K_NP_*, both the maximum AGB and BGB in Scenarios 4–5 were significantly higher than in Scenario 1. Conversely, they were lower in Scenarios 2–3 than in Scenario 1. This pattern indicated the facilitating effect of *K_NP_* on the growth of *P. australis*, which was consistent with the findings in several other cases (e.g., Burullus Lake, Egypt; Swazaro, Poland) [71,72]. Subject to varying *K_NP_*, new rhizomes began to grow at different rates after *t_p_* (the date when peduncles and new rhizomes start forming) because of different amounts of current photosynthesized material transferred to the below-ground organs, leading to contrasting biomass in the below-ground part. After *t_s_* (the date when shoots start senescence), the senescence of the above-ground organs led to abrupt decline in the AGB and also diminished the contrast among the various scenarios. At the same time, the transport of photosynthetic materials from the above-ground part to the below-ground part widened the gap in the BGB. This is also consistent with the finding reported by Karunaratne et al. [59] that increased nutrient availability leads to higher living biomass as well as more photosynthetic materials transported to the below-ground part.

**Figure 9:**
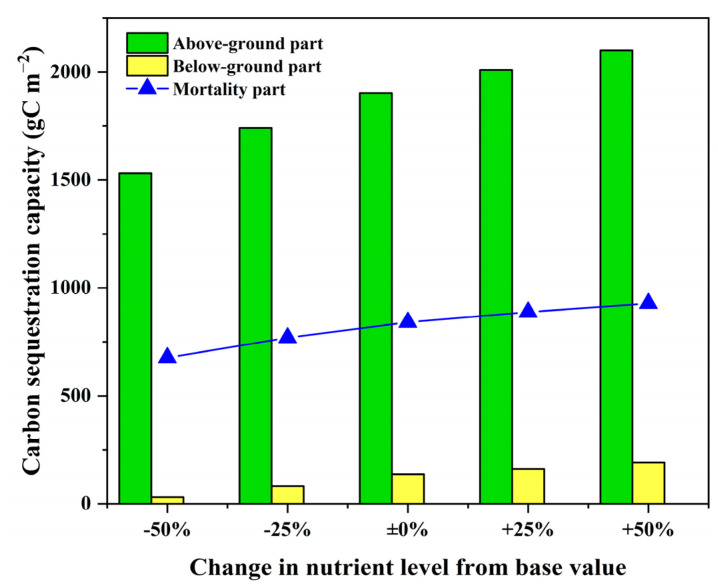
Annual carbon sequestration capacity in above- and below-ground and mortality parts of *P. australis* growing with different nutrient availability.

As a result of the biomass change, our results show that the annual carbon sequestration capacity increases by 12% when *K_NP_* increases by 50%, and decreases by 23% when *K_NP_* decreases by 50% (Figure 9); i.e., annual carbon sequestration capacity of *P. australis* is enhanced by a higher *K_NP_* [71,72,75,76]. Hence, comprehensive analysis of the ecological consequences of nutrient removal and carbon sequestration capacity should be performed in future wetland management to optimize the trade-off between eutrophication control and carbon sequestration. Additionally, as shown in Section 2.5, carbon sequestration was estimated based on the contribution of both living parts (i.e., above- and below-ground part) and the mortality part in the present study. Our results show that the magnitude of carbon sequestration capacity of each part had a positive correlation with increasing *K_NP_* (Figure 9), which was consistent with reported findings from other studies [77]. However, the effect of the decomposition of mortality part was not considered in our carbon sequestration analysis. In fact, this process can trigger the carbon release and cause loss of carbon sequestration capacity of *P. australis* [77,78]. As the *K_NP_* increases, enhanced carbon sequestration capacity from the mortality part can cause larger loss in carbon sequestration capacity if not treated properly. As such, we recommend that more frequent harvest and regular collecting of mortality organs should be carried out [77,79] after the senescence begins so that carbon sequestration loss due to decomposition can be reduced.

## 5. Conclusions

In this study, an existing growth dynamics model was adapted to estimate biomass and carbon sequestration capacity of *P. australis* during a typical growth cycle in the Hanshiqiao Wetland Nature Reserve (HWNR) in Beijing, China. The AGB retrieved from hyperspectral images of the study area was used for model calibration. The results show that the AGB reached its peak of 2930 g/m^−2^ in late August and that the maximum BGB of 2486 g m^−2^ is observed in early November. The maximum monthly carbon sequestration capacity is attained in July, and the annual carbon sequestration capacity is 2040 gC m^−2^ yr^−1^, which translates to 1766 tC to be sequestrated by *P. australis* growing in the HWNR. In addition to latitude, inundation depth and nutrient level are found to have significant effects on the growth of *P. australis* in the HWNR and account for its relatively high biomass compared with nearby sites with similar latitudes. Scenarios with varying nutrient availability were also simulated, and the results show that nutrient availability has a facilitating effect on the growth and carbon sequestration capacity of *P. australis* in the HWNR. Based on the modelling results, in future practice, we recommend the comprehensive management of urban wetlands that often encounter eutrophication problems to synergize the effects of nutrient control and carbon sequestration. In addition, it is notable that relative to living parts (above- and below-ground part), the contribution of mortality part to carbon sequestration was disproportionately enhanced by increasing nutrient availability, leading to enlarged potential loss of carbon sequestration capacity due to decomposition of mortality organs. Therefore, more frequent harvest and regular collecting of the mortality part of *P. australis* should be carried out after the senescence begins to reduce the carbon sequestration loss.

The zero-dimensional plot-scale plant growth dynamics model is useful for a preliminary estimation of the plant biomass and carbon sequestration capacity throughout the vegetated area in a wetland. A spatially explicit model to account for spatial heterogeneity in plant growth can be developed and applied in the future. The empirical retrieval model established by Li et al. [33] was based on field biomass data gathered in 2017, and interannual variation may lead to some uncertainty in our application. We only consider the contribution of plant photosynthesis in our carbon sequestration estimation, and other relevant processes in the wetland carbon cycle such as microbial decomposition and respiration in soil [80] can be incorporated in future ecosystem carbon sequestration estimation.

## Figures and Tables

**Table 1:** Main parameters of Orbita hyperspectral satellite constellation (OHS).

Parameter	Value
Spatial resolution	10 m
Swath width	150 × 150 km
Satellite mass	67 kg
Sensor height	500 km
On-orbit life span	>5 years
Signal-to-noise ratio	>30
Wavelength	400 nm to 1000 nm
Spectral resolution	2.5 nm
Bands	256 (32 bands can be selected)
Revisit cycle	5 days

**Table 2:** The major phenological points and the corresponding phenological events.

Phenological Points	Phenological Events
*t_r_*	The formation of new roots and fertile leaves, as well as the beginning of translocation of dry matter from old rhizomes to fertile leaves and roots
*t_b_*	The formation of sterile leaves and non-flowering secondary shoots, as well as the beginning of translocation of dry matter from old rhizomes to sterile leaves and non-flowering secondary shoots
*t_p_*	The formation of new rhizomes and peduncles, together with the beginning of translocation of dry matter from old rhizomes to peduncles, as well as the beginning of translocation of photosynthesized material to below-ground plant organs
*t_f_*	The appearance of panicles as well as the beginning of translocation of dry matter and photosynthesized material from peduncles to panicles
*t_e_*	The ending of mobilization of dry matter from rhizomes to shoots and roots
*t_s_*	The commencement of shoot senescence, together with the beginning of translocation of accumulated shoot dry matter to below-ground organs

**Table 3:** Training and validation samples from the high-resolution BJ-2 image.

Parameter	Training Samples	Validation Samples	Total
Water body	74	32	106
*P. australis*	59	33	92
*N. tetragona*	14	10	24
*T. orientalis*	15	11	26
Other vegetation	75	21	96

**Table 4:** Sample separability between *P. australis* and other classes.

Class	JM Value	TD Value
Water body	1.97	1.99
*N. tetragona*	1.99	2
*T. orientalis*	1.99	2
Other vegetation	1.98	1.99

**Table 5:** Interpretation accuracies.

Class	Producer’s Accuracy (PA)	User’s Accuracy (UA)
Water body	87.67%	82.40%
*P. australis*	94.71%	96.95%
*T. orientalis*	70.89%	81.05%
*N. tetragona*	75.87%	71.10%
Other vegetation	94.45%	94.78%
Overall accuracy	92.21%	
Kappa coefficient	0.88	

**Table 6:** Sensitivity of model predictions on key modeling parameters.

**Parameters**	**Variation (%)**	**AGB (%)**
Maximum specific net daily photosynthesis rate	+50	+56.35%
	−50	−58.01%
Specific mortality rate of shoots	+50	−17.14%
	−50	+21.46%
Fraction of shoot transfer to rhizome	+50	−12.41%
	−50	+14.73%
Constant of the availability of nutrients	+50	+8.95%
	−50	−17.38%
Fraction of shoot biomass for elongation	+50	+7.08%
	−50	−13.48%

**Table 7:** The phenological and growth traits of *P. australis* in various study sites from different latitudes.

Parameter	Region				
	AR	HWNR	NFP	LB	VNR
Latitude	35°51′ N	40°06′ N	48°48′ N	56°65′ N	57°05′ N
Primary shoot growth start (J-day)	93	140	110	83	100
Panicle appearance (J-day)	213	225	196	213	232
Maximum above-ground biomass (g/m^2^)	3379.6	2930.1	2050	669	1145.6

AR: Arakawa River in Saitama City, Japan [59]; LB: Loch of Balgavies, Scotland [67]; NFP: Nesyt fish pond, Czech Republic [69]; VNR: Vejlerne Nature Reserve, Denmark [68].

## Data Availability

Not applicable.

## Supplementary Materials

The following supporting information can be downloaded at: https://www.mdpi.com/article/10.3390/s22093141/s1, Figure S1: Ground-truth samples of ground objects; Table S1: Parameters used in the growth dynamics model; Table S2: Total annual biomass of mortality of *P. australis* growing with different nutrient availability; Equations (S1)–(S17): Equations used to simulate the dynamic growth of *P. australis* in study site.
