# Supplementary material for: Estimating Biomass and Carbon Sequestration Capacity of Phragmites australis Using Remote Sensing and Growth Dynamics Modeling: A Case Study in Beijing Hanshiqiao Wetland Nature Reserve, China
Source: Sensors (Basel). 2022 Apr 20;22(9):3141. doi: 10.3390/s22093141 (PMC9105711; doi:10.3390/s22093141)
Supplement: Supplementary file 1 [file sensors-22-03141-s001.zip › sensors-1668579-supplementary.pdf]

1. Figure

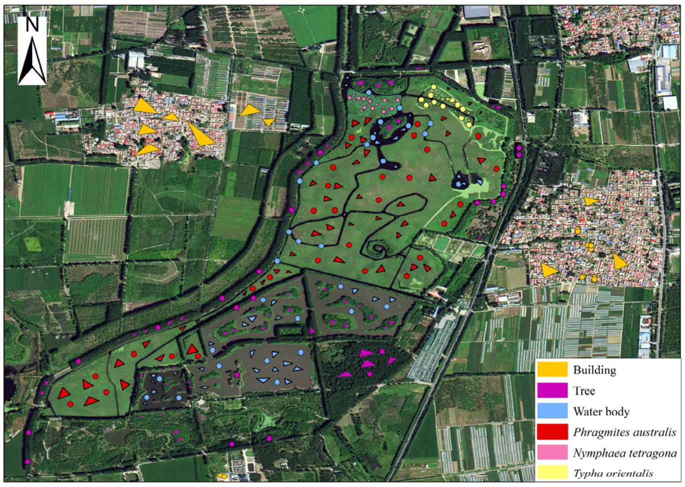

Figure S1. Ground-truth samples of ground objects, including building, tree, water body, *Phragmites australis*, *Nymphaea tetragona*, and *Typha orientalis*, where triangles and circles represent training samples and validation samples, respectively.

2. Table

Table S1. Parameters used in the growth dynamics model.

Parameter	Notation
Maximum specific growth rate of roots at 20 ℃	g_m
Specific dark respiration rate of shoots at 20 ℃	β_{msht}
Specific respiration rate of roots at 20 ℃	β_{rt}
Specific respiration rate of old rhizomes at 20 ℃	β_{rhi}
Specific respiration rate of new rhizomes at 20 ℃	R_n
Specific respiration rate of panicles at 20 ℃	R_p
Specific mortality rate of shoots from t_b to t_p at 20 ℃	γ_{sht}
Specific mortality rate of shoots from t_p to t_s at 20 ℃	γ_{sht}
Specific mortality rate of shoots after t_s at 20 ℃	γ_{sht}
Specific mortality rate of panicles from t_p to t_s at 20 ℃	γ_p
Specific mortality rate of panicles after t_s at 20 ℃	γ_p
Specific mortality rate of roots at 20 ℃	γ_{rt}
Specific mortality rate of old rhizomes at 20 ℃	γ_{rhi}
Specific mortality rate of new rhizomes at 20 ℃	γ_n
Fraction of current photosynthesis translocation to below ground structures	ε_{ph}
Fraction of shoot assimilates translocation to below ground structures	ε_{sht}
Fraction of shoot assimilates translocated for old rhizomes	y
Fraction of shoot assimilates translocation for inflorescence	p
Fraction of current photosynthesis translocation to inflorescence	k
Fraction of shoot biomass for elongation	q
Fraction mobilized from rhizomes for root formation	x
Maximum specific net daily photosynthesis rate of at 20 ℃	P_m
Half saturation constant of age for shoot photosynthesis	K_{age}
Half saturation constant of *PAR* for shoot photosynthesis	K_{PAR}
Half saturation constant of age for root growth	K_{rt}
Temperature constant	θ
Conversion constant of carbon dioxide to ash-free dry weight	k_m
The date when roots and flowering shoots start growing	t_r
The date when non-flowering shoots start growing	t_b
The date when peduncles and new rhizomes start forming	t_p
The date when panicles start forming	t_f
The date when the upward translocation from old rhizomes terminates	t_e
The date that marks the onset of senescence	t_s

Table S2. Total annual biomass of mortality of *P. australis* growing with different nutrient availability.

**Changes in nutrient level**	–50%	–25%	±0%	+25%	+50%
**Biomass of mortality (g m^{-2})**	3419.48	3933.002	4272.437	4532.113	4724.532

3. Equation

In this section we document the equations used to simulate the growth dynamics of *P. australis*. Phenological points used in these equations, including t_r, t_b, t_p, t_f, t_e and t_s, are illustrated in Section 2.3.1 of the main text.

Equations S1–S5 show the net growth of each organ of *P. australis*, where the subscript rhi, rt, n, sht, and p represent old rhizomes, roots, new rhizomes, shoots, and panicles, respectively.

$$\frac{\partial B_{\mathrm{rhi}}}{\partial t} = -R_{\mathrm{rhi}} - D_{\mathrm{rhi}} - \mathrm{Rhif} \times f_{\mathrm{rhi}} + y \sum_{i=1}^{i=i\max} \varepsilon_{\mathrm{sht}} b_{\mathrm{sht}}(i) f_{\mathrm{sht}} + y \sum_{i=1}^{i=i\max} \varepsilon_{\mathrm{ph}} \mathrm{Ph}_{\mathrm{sht}}(i) f_{\mathrm{ph}} \quad \text{(S1)}$$

$$\frac{\partial B_{\mathrm{rt}}}{\partial t} = G_{\mathrm{rt}} f_{\mathrm{rt}} - R_{\mathrm{rt}} - D_{\mathrm{rt}} + xR\mathrm{hif} \times f_{\mathrm{rhi}} \quad \text{(S2)}$$

$$\frac{\partial B_{\mathrm{n}}}{\partial t} = -R_{\mathrm{n}} - D_{\mathrm{n}} + (1-y) \sum_{i=1}^{i=i\max} \varepsilon_{\mathrm{sht}} b_{\mathrm{sht}}(i) f_{\mathrm{sht}} + (1-y) \sum_{i=1}^{i=i\max} \varepsilon_{\mathrm{ph}} b_{\mathrm{ph}}(i) f_{\mathrm{ph}} \quad \text{(S3)}$$

$$\frac{\partial b_{\mathrm{sht}}(i)}{\partial t} = \mathrm{Ph}_{\mathrm{sht}}(i) - R_{\mathrm{sht}}(i) - D_{\mathrm{sht}}(i) + (1-x)\mathrm{Rhif} \times f_{\mathrm{rhi}}\left(\frac{b_{\mathrm{sht}}(i)}{B_{\mathrm{sht}}}\right) - \varepsilon_{\mathrm{sht}} b_{\mathrm{sht}}(i) f_{\mathrm{sht}}$$
$$-\varepsilon_{\mathrm{ph}} \mathrm{Ph}_{\mathrm{sht}}(i) f_{\mathrm{ph}} - (b_{\mathrm{sht}}(i)\varepsilon_{\mathrm{p}} + \mathrm{Ph}_{\mathrm{sht}}(i)k) ff - G_{\mathrm{rt}} f_{\mathrm{rt}}\left(\frac{b_{\mathrm{sht}}(i)}{B_{\mathrm{sht}}}\right) \quad \text{(S4)}$$

$$\frac{\partial B_{\mathrm{p}}}{\partial t} = -R_{\mathrm{p}}(i) - D_{\mathrm{p}}(i) + \sum_{i=1}^{i=i\max} \mathrm{Ph}_{\mathrm{sht}}(i) k \times ff + \sum_{i=1}^{i=i\max} \varepsilon_{\mathrm{p}} b_{\mathrm{sht}}(i) ff \quad \text{(S5)}$$

For Equation S1, R_{rhi} and D_{rhi} are the specific rates of respiration and mortality, respectively, at 20 ℃ for old rhizomes; Rhif is the mobilization of stored material from rhizomes to roots and shoots during the initial stage of growth; f_{rhi} assumes unity for $t_b \le t \le t_e$ and 0 otherwise; $b_{\mathrm{sht}}(i)$ is the shoot biomass of layer i; $\varepsilon_{\mathrm{sht}}$ is the fraction of shoot assimilating translocation to below-ground structures; f_{sht} assumes unity for $t > t_s$ and 0 otherwise; $\varepsilon_{\mathrm{ph}}$ is the fraction of current photosynthesis translocation to below-ground structures; $\mathrm{Ph}_{\mathrm{sht}}(i)$ represents the shoot biomass of layer i accumulated by photosynthesis, which can be calculated using Equation S11; f_{ph} assumes unity for $t_p \le t \le t_s$ and 0 otherwise.

For Equation S2, G_{rt} is the supply of photosynthesized material for root growth, which can be calculated using Equation S10; f_{rt} assumes 1 for $t_r \le t \le t_p$ and 0 otherwise; R_{rt} and D_{rt} represent the specific rates of respiration and mortality, respectively, at 20 ℃ for roots; x is the fraction mobilized from

rhizomes for root formation.

For Equation S3, R_n and D_n represent the specific rates of respiration and mortality, respectively, at 20 ℃ for new rhizomes; y is the fraction shoot assimilates translocated for old rhizomes.

For Equation S4, $R_{sht}(i)$ and $D_{sht}(i)$ represent the specific rates of respiration and mortality, respectively, at 20 ℃ for shoots in layer i; f_{sht} assumes 1 for $t > t_s$ and 0 otherwise; k and ε_p are the fractions of contribution of the current photosynthesis and accumulated shoot dry matter to the formation of panicles; ff assumes 1 for $t_f \leq t$ and 0 otherwise.

For Equation S5, $R_p(i)$ and $D_p(i)$ represent the specific rates of respiration and mortality, respectively, at 20 ℃ for panicles in layer i.

Rhif, R_a, D_a, G_{rt}, $\mathrm{Ph}_{sht}(i)$ in Equations S6–S15 were calculated as follows:

$$\mathrm{Rhif} = \alpha_{rhi}\theta^{(T-20)}B_{rhi} \quad \text{(S6)}$$

$$\alpha_{rhi} = 0.58(\text{initial rhizome biomass})^{-0.50} \quad \text{(S7)}$$

where α_{rhi} is the specific transfer rate of rhizome biomass; θ (=1.09) is the temperature constant; T stands for the average daily temperature.

$$R_a = \beta_a\theta^{(T-20)}B_a \quad \text{(S8)}$$

$$D_a = \gamma_a\theta^{(T-20)}B_a \quad \text{(S9)}$$

where γ_a and β_a are the specific rates of respiration and mortality, respectively, at 20 ℃; subscript a represents old rhizomes, new rhizomes, roots, panicles, or shoots.

$$G_{rt} = \mathrm{g_m}\theta^{(T-20)}\frac{K_{rt}}{K_{rt} + \mathrm{Age}_{rt}}B_{rt} \quad \text{(S10)}$$

where $\mathrm{g_m}$ is the maximum specific growth rate of roots at 20 ℃; K_{rt} is the half saturation coefficient of root age; Age_{rt} is the age of roots in days from the start of root growth; B_{rt} represents the biomass of roots.

$$\mathrm{Ph}_{sht}(i) = P_m k_{co}\theta^{(T-20)}\frac{I_{PAR}(i)}{K_{PAR} + I_{PAR}(i)} \times \frac{K_{age}}{K_{age} + \mathrm{Age}_{sht}}b_{sht}(i) \quad \text{(S11)}$$

where P_m is the maximum specific net daily photosynthesis rate of the plant top at 20 ℃ in the absence of light and nutrient limitations; k_{co} is the

conversion constant of carbon dioxide to ash-free dry weight; $\mathrm{Age}_{\mathrm{sht}}$ is the age of shoots from the start of growth; K_{PAR} and K_{age} are the half saturation coefficients of photosynthetically active radiation (PAR) and age, respectively. $I_{\mathrm{PAR}}(i)$ is the photosynthetically active radiation in the ith layer of shoot, which reads

$$I_{\mathrm{PAR}} = 0.45 \times \text{global radiation} \tag{S12}$$

$$I_{\mathrm{PAR}}(i) = I_{\mathrm{PAR}} e^{-kF_i} \tag{S13}$$

where I_{PAR} is the PAR in the open, and F_i is the cumulative leaf area index, in which F_i reads

$$F_i = \sum_{i=1}^{i=i} \mathrm{LAI}(i) \tag{S14}$$

where LAI is the leaf area index and was calculated as follows:

$$\mathrm{LAI}(i) = 0.01335[b_{\mathrm{leaf}}(i)]^{1.02} \tag{S15}$$

where $b_{\mathrm{leaf}}(i)$ is the leaf biomass in the ith layer of shoot.

The regression relationship between phenological points in this study are shown as follows:

$$(t_e - t_b) = 0.14(t_f - t_b)^{1.27} \tag{S16}$$

$$(t_s - t_b) = 0.22(t_f - t_b)^{1.36} \tag{S17}$$
